# Suitability Study of Optical Coordinate Measuring Machine for Quality Assessment and Wear Phenomena Identification of Blade Edge and Surface of Planer Technical Knives

**DOI:** 10.3390/ma17164018

**Published:** 2024-08-13

**Authors:** Magdalena Rzepka, Czesław Łukianowicz, Wojciech Zawadka, Krzysztof Rokosz, Krzysztof Nadolny

**Affiliations:** 1 Department of Production Engineering, Faculty of Mechanical and Energy Engineering, Koszalin University of Technology, Racławicka 15-17, 75-620 Koszalin, Poland; magdalenaa.rzepkaa@gmail.com (M.R.); czeslaw.lukianowicz@tu.koszalin.pl (C.Ł.); wojciech.zawadka@tu.koszalin.pl (W.Z.); krzysztof.nadolny@tu.koszalin.pl (K.N.); 2 Faculty of Electronics and Computer Science, Koszalin University of Technology, Śniadeckich 2, 75-453 Koszalin, Poland

**Keywords:** planer knives, edge and surface wear, optical coordinate machine

## Abstract

This article discusses a comparative analysis of the wear and quality of planer knife blades used in wood planers. The novelty in this work is the use of a simple coordinate measuring machine with a vision system to assess the wear of the cutting edges of planer knives. The primary objective of the research described in this paper was to verify whether the wear of the cutting edge of planer knives can be measured quickly and accurately using an optical coordinate measuring machine with a vision system. To date, contact profilometry methods have been used for this purpose, which require a specialist apparatus and qualified measuring equipment operators and are expensive and time-consuming. The research presented in this work was conducted on twelve planer knives. The condition and wear of the working surfaces of the tested knives were assessed using an optical digital microscope. The wear of the cutting edge of the knives was measured using two methods: the contact profilometry method and an optical coordinate measuring machine equipped with a vision system. The edge profiles and their parameters obtained by the optical method were compared to the results of measurements with a stylus profilometer. Based on the research and analyses conducted, it was found that the optical method used in this research significantly shortens the time of measuring the wear of the cutting edges of planer knives. In addition, this method has a wider measurement range, and the obtained measurement results are characterized by lower measurement uncertainty.

## 1. Introduction

In various industries, machines and tools are crucial for carrying out technological operations that involve material processing. Technical blades are tools used for cutting and incising materials in processes within the wood, paper, food, textile, and chemical industries. Most often, the blades come in the form of replaceable knives or cutting inserts and significantly impact production processes. The quality of technical blades is primarily evaluated based on their functional properties, including cutting ability and wear resistance. These properties depend on the blade material, dimensions, and shape [1,2,3]. The selection of these features and parameters is influenced by the specific purpose of the blade and the material being cut. Technical knives and blades are typically constructed from carbon tool steels, high-speed alloy steels, and stainless steels, including materials such as tungsten carbide and sintered ceramics like zirconium oxide (ZrO_2_).

Often, to increase the durability of technical blades made of HSS steel and sintered carbides, i.e., the wear resistance of their surfaces and cutting edges, they are subjected to thermochemical treatment or covered with special coatings that increase abrasion resistance and reduce the friction coefficient. Technical blades with increased wear resistance are sharpened or replaced less frequently during operation, which shortens the downtime of technological devices related to their sharpening or replacement and helps to grow production efficiency [4,5,6,7]. Such tools require the use of appropriate regeneration processes during their operation.

The woodworking industry uses combined surface and thickness planing machines [8,9]. To ensure the proper quality of products, it is necessary to monitor the condition and durability of the tools used in these machines and assess their wear. Regarding planer technical knives, this assessment includes analysing the texture and conditions of the rake surface of knives and measuring the so-called edge recession, which is the edge’s displacement in the cutting knife’s working section due to wear [7,10,11,12].

The micro-geometry of the cutting edge depends on the way it is shaped and changes significantly because of wear processes during blade operation [13]. The contact profilometry method is the most frequently used way to directly assess the wear of cutting edges. This method allows for the assessment of the plastic deformations of the cutting edges of blades, caused, among others, by tribological wear accompanying operational processes. Contact stylus methods, used mainly to assess the geometric structure of surfaces, are widely described in the literature [14,15]. In addition to direct methods for assessing blade edge wear, indirect methods are also used, which are based on the assessment of the sharpness [1,3], durability [16], cutting forces [17,18], or cutting ability of the blade [19]. In the precision industry, in recent years, there has also been a tendency to assess the condition of the edges and working surfaces of cutting tools directly on machine tools. For this purpose, various contact and advanced optical methods are also used [20,21].

A quantitative analysis of the wear and quality of planer knives is performed using various methods. Most often, these consist of the use of contact profilometric methods or various optical systems. In works [7,10], the use of contact and optical profilometers for this purpose was shown, thanks to which the wear of the cutting edge and working surfaces of planer knives was assessed after intensive use. Similar studies were conducted to determine the durability of anti-wear coatings applied to the working surfaces of planer knives [22,23].

For the assessment of the wear of other tools, such as milling cutters, drills, lathe tools, etc., optical methods are most often used, based on vision systems and machine vision systems [24,25,26]. The creation of such systems is dictated by the desire to measure during tool operation and most often requires complex software for image processing, analysis, and interpretation [27]. Universal coordinate measuring systems, including optical systems, are rarely used to measure the wear of typical cutting tools. In this work, a coordinate optical measuring system was used to assess the wear of planer knife edges. The obtained results were compared with the results of contact profilometric measurements.

**Figure 1:**
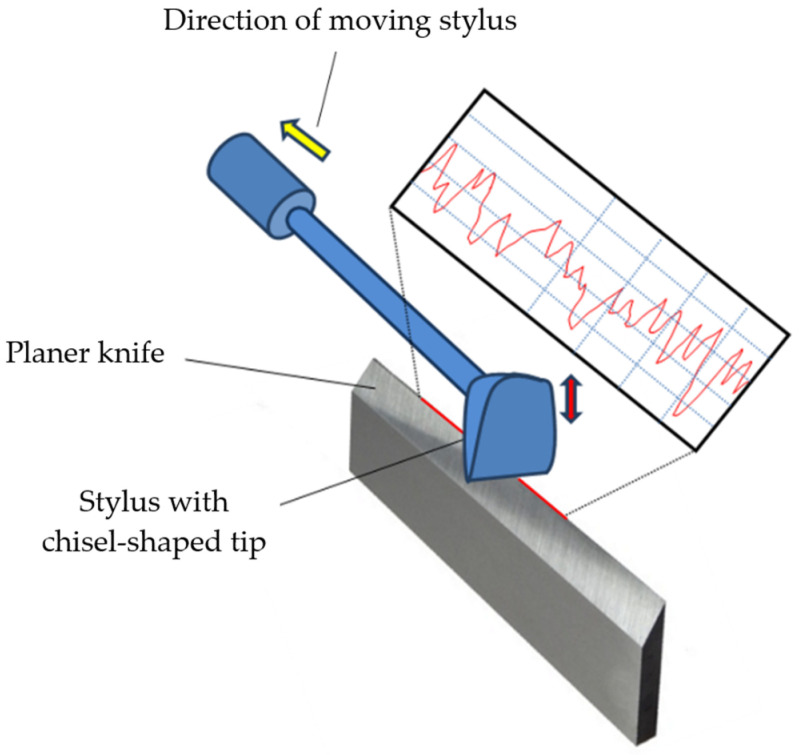
The principle of measuring the edge profile of planer knives during wear tests using a contact profilometer equipped with a chisel-shaped stylus.

Adapting contact profilometry measurements to assess the wear of the edges of technical blades requires equipping the profilometer with an unusual mapping stylus with a chisel-shaped tip (as shown in Figure 1) because the set of points that make up the actual edge of the blade is a three-dimensional object. Moreover, due to the contact profilometry measurements of knife edge wear, the projection of the three-dimensional real edge is only determined on the plane in which the measurement was made. In practical applications of contact profilometry methods, the time needed to perform such an assessment is also important. For blades several dozen millimetres long, the measuring process takes at least several minutes and increases in duration with the blade length. A similar method was used, among others, in works [7,10].

In research carried out over the last few years at the Koszalin University of Technology, the contact method was used to determine the wear profile of the edges of planer knife blades [7,10,11]. In these studies, the contact profilometry method was used to assess the wear of the cutting edges of planer knives working in the heads of wood planers. For research purposes, this method turned out to be useful. However, the use of contact measurements in production conditions has not been accepted, mainly due to the relatively long time needed to assess edge wear and the complex procedures for preparing and implementing the measurement process. Therefore, it was decided to consider an alternative solution for the wear assessment of the cutting edges of planer knives, which would simplify the measurement methodology and shorten the assessment time. Instead of contact profilometric measurements to determine the wear profile of the cutting edges of planer knives, it was proposed to use an optical coordinate measuring machine (OCMM) for this purpose. This article presents comparative studies of both methods of measuring and assessing the wear of the cutting edges of planer knives.

## 2. Materials and Methods

### 2.1. Purpose and Subject of Research

The aim of this research was to conduct a comparative assessment of the suitability of an OCMM for assessing the quality and wear of the blade edges and working surfaces of planer knives. Initially, using a digital microscope, images of selected fragments of the rake surface of the tested planer knives were recorded and analysed. For each knife, images of worn and unworn (reference) fragments of the rake surface were recorded. Basic tests were carried out in two stages on 12 planer knives. In the first stage, the wear of the cutting edges of the knives was examined using the contact profilometry method. In the second stage, similar tests were carried out using the OCMM. Then, the research results obtained using both methods were compared and discussed.

### 2.2. The Characteristics of the Tested Planer Knives

**Figure 2:**
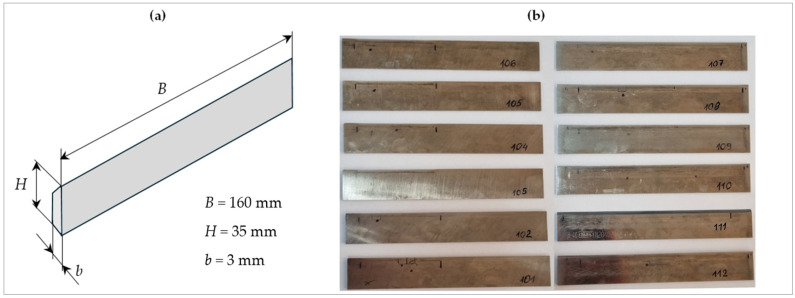
Tested planer knives: (**a**) dimensions of tested planer knives and (**b**) view of knives marked with symbols NN101 to NN112.

As mentioned earlier, twelve planer knives, marked for research purposes with symbols NN101 to NN112, with nominal dimensions 160 × 30 × 3 mm from FABA LLC (Baboszewo, Poland), were selected for testing. The knives were made of tungsten–molybdenum high-speed steel HS6-5-2. The basic material characteristics of HS6-5-2 steel are presented in Table 1. The knives were not coated with anti-wear layers. A view of the tested planer knives is shown in Figure 2.

The tests on planer knives were preceded by their operation on the production line. The knives were installed in the planer heads of the Hydromat 22B planer from Michael Weinig AG (Tauberbischofsheim, Germany) used in a company producing wooden elements. The wear of the cutting edges and working surfaces of the knives appeared as an effect along the work of the planer heads on the production line. Knives with numbers NN101 to NN106 were used to process wooden elements with a width of approximately 65 mm. To process elements with a width of approximately 148 mm, knives with numbers NN107 to NN112 were used.

After machining, the planer knives were removed from the planer heads. Each knife was thoroughly cleaned of dust, chips, and other contaminants. The planer knives prepared in this way were subjected to microscopic examination. Then, the wear of the cutting edges was assessed, first using a contact profilometer and then the OCMM. After the wear tests, the planer knives were sharpened and returned to the production line.

### 2.3. Research Stands and Methodology of Experimental Research

**Figure 3:**
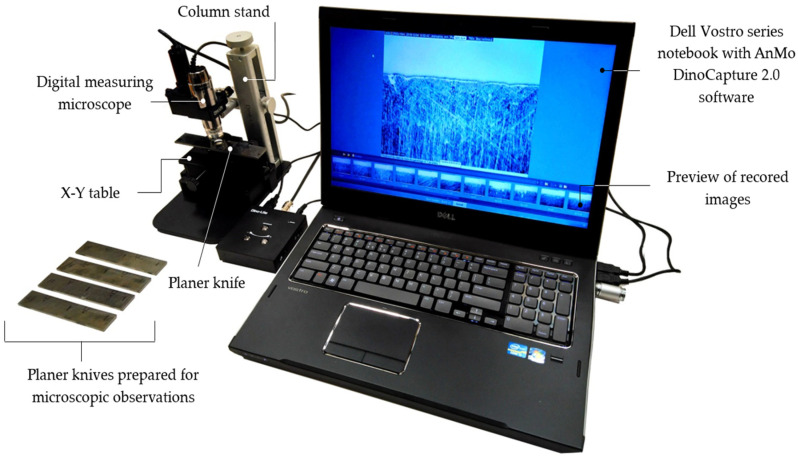
A view of the stand for acquiring, testing, and analysing digital images of the working surfaces of planer knives equipped with a digital measuring microscope Dino-Lite Edge AM755MT8A.

Qualitative tests of the edges and the rake surfaces of planer knives were conducted on a test stand intended to be used to acquire, record, and analyse the digital microscopic images. The stand was equipped with a high-resolution Dino-Lite Edge AM7515MT8A digital microscope from AnMo Electronics Corp. (New Taipei City, Taiwan). The microscope’s optical system allows for image acquisition at magnifications ranging from 700 to 900× and the recording of video sequences at 30 frames per second. The matrix image detector of this microscope uses complementary metal–oxide–semiconductor (CMOS) technology and enables the recording of images with a resolution of 5 Mpx (2592 × 1944 pixels). The microscope was powered by the USB port of a Vostro series notebook from Dell (Austin, TX, USA). Figure 3 shows a general view of the stand for observing and microscopic tests of planer knives.

The microscope was equipped with a light source containing 8 integrated light-emitting diodes (LEDs) with the flexible LED control (FLC) function of flexible lighting intensity control. It allowed for switching between bright field and coaxial lighting or combining them. Concentric lighting technology is particularly beneficial because it reveals details that are difficult to see under normal conditions. The microscope had an automatic magnification reading (AMR) function that detected the magnification and displayed it in the Dino-Lite Capture 2.0 software.

**Figure 4:**
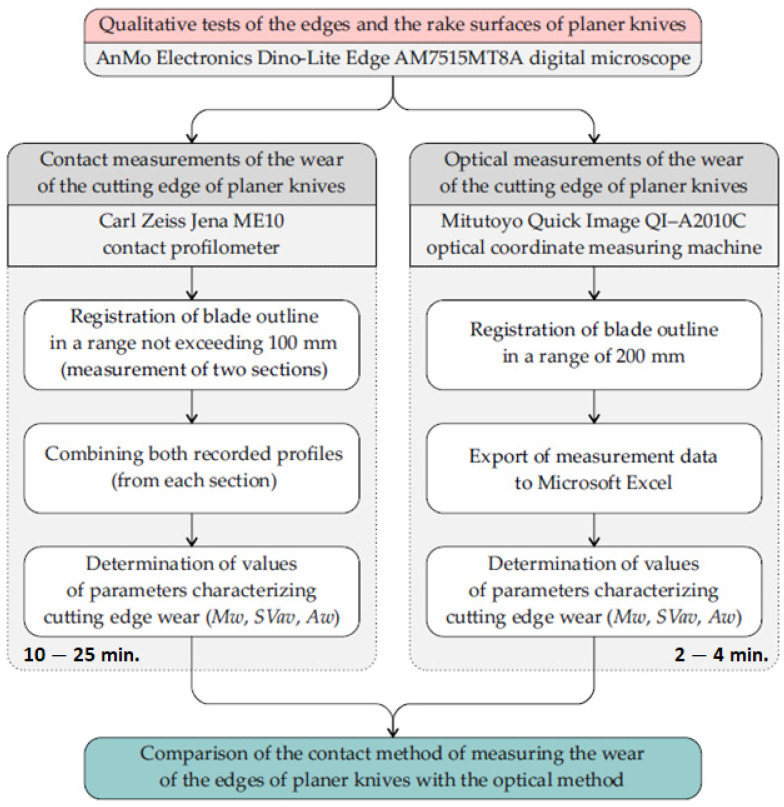
A flowchart of the experimental research process.

A flowchart of the experimental research process is shown in Figure 4.

**Figure 5:**
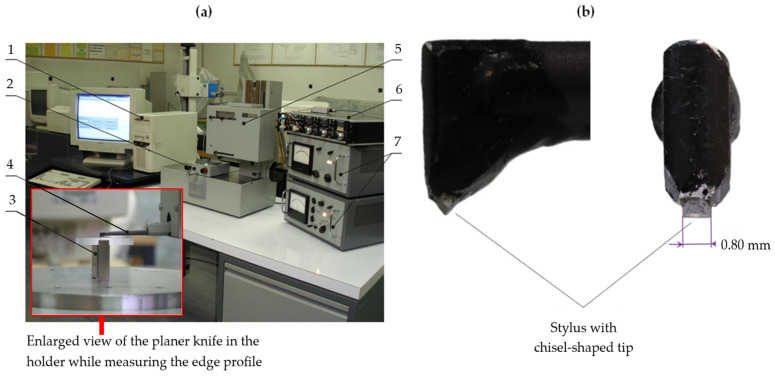
The test stand for the contact profilometry measurements of the cutting edges of planer knives: (**a**) the general view of the stand; 1—a computer with a data acquisition card, 2—object table, 3—holder with a knife, 4—arm of the profilometer measuring head ending with a stylus, 5—profilometer drive unit, 6—power supply, 7—processing system; (**b**) enlarged side and frontal views of a stylus with a chisel-shaped tip.

Contact measurements of the wear of the cutting edge of planer knives were carried out using a modernized ME10 contact profilometer manufactured by Carl Zeiss Jena (Jena, Germany). It was equipped with a stylus with a chisel-shaped tip. A view of the test stand for the contact profilometry measurements of the cutting edges of planer knives with the ME10 profilometer is shown in Figure 5a. In addition, Figure 5b shows an enlarged side and frontal view of a fragment of a stylus with a chisel-shaped tip.

Since the test stand with the ME10 profilometer enabled the registration of the outline in a range not exceeding 100 mm, when testing knives with numbers NN107 to NN112, the length of which as well as the width of the wear section exceeded this value, the measurements had to be carried out twice, and then both recorded profiles were combined. This significantly increased the measurement and analysis time, because only after combining both knife edge profiles were the values of the parameters characterizing wear determined.

**Figure 6:**
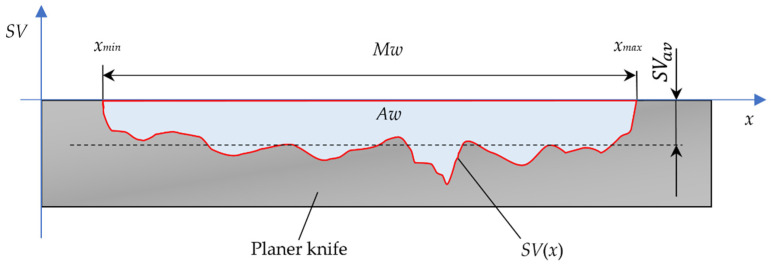
A diagram showing the assessed wear parameters of the edge of a planer knife: *Mw*—machining width, *SVav*—average value of worn edge displacement, and *Aw*—wear area of the cutting edge.

Using the contact method, a methodology described in [7,10] was used to test the wear of planer knife profiles. The parameters characterizing the wear of knives were assessed based on the recorded wear profiles of the cutting edges of planer knives. This is shown in the diagram in Figure 6. For each planer knife, the width of the wear zone *Mw*, the average depth of wear of the knife edge *SVav*, determined over the entire width of the blade wear section, and the wear area cutting edge planer knife *Aw* were determined. Calculations were made based on the following relationships:

(1) $$Mw=x_{max}-x_{min},$$

(2) $${SV}_{av}=\left|\frac{1}{n}\sum_{x_{min}}^{x_{max}}\;SV\left(x\right)\right|,\;\;\mathrm{for}\;n=\frac{Mw}{\Delta x}\;$$

(3) $$Aw=0.001\;Mw\cdot {SV}_{av},$$

where *Mw*—machining width (width of the wear zone of the cutting edge planer knife), mm; *xmin*—abscissa of the beginning of the wear section, mm; *xmax*—abscissa of the end of the wear section, mm; *SVav*—average value of worn edge displacement, µm; *SV*(*x*)—worn edge displacement at point *x*, µm; *n*—number of samples in the wear zone; *Δx*—sampling step, mm; *Aw*—wear area of the cutting edge, mm^2^.

The time for measuring the knife edge profile with a contact profilometer for planer knives numbered from NN101 to NN106 was approximately 10 min. However, for knives ranging from NN107 to NN112, the measurement time was more than twice as long and often amounted to 25 min. The signal from the contact profilometer representing the profile of the knife edge in digital form was recorded on a computer. The signal sampling step *Δx* was 0.040 mm. In the case of six planer knives numbered NN101 to NN106, the data files contained approximately 1700 values. However, for the remaining six knives numbered NN107 to NN112, the data files contained approximately 3800 values. These files were processed using a program developed in a Microsoft Excel spreadsheet. The program allowed for the preparation of edge profile charts of the tested knives and the determination of the values of the *Mw*, *SVav*, and *Aw* parameters characterizing the wear of the cutting edges of the knives.

The decision was made to compare the contact method of measuring the wear of the edges of planer knives with the optical method. This comparison involved considering various optical techniques, such as optical coordinate measuring machines (OCMMs) [28,29,30,31], for this purpose.

**Figure 7:**
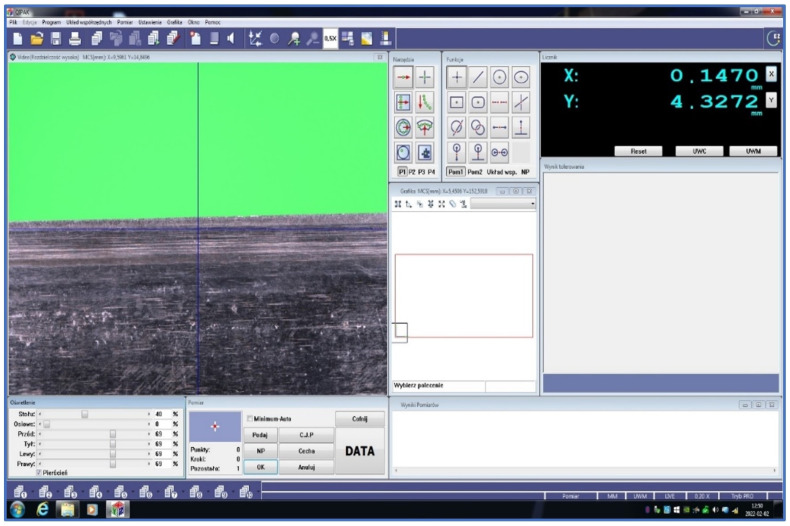
A view of the OCMM Quick Image QI–A2010C screen during preparation for measuring the wear of the cutting edge of the planer knife.

**Figure 8:**
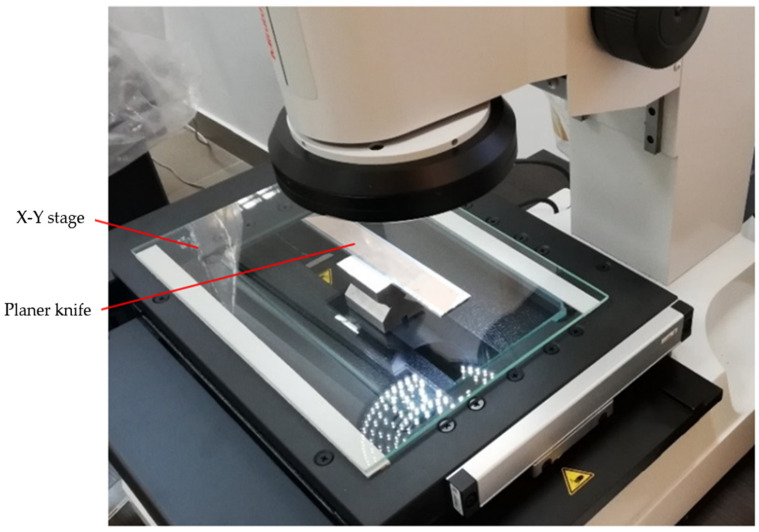
A view of the Quick Image QI–A2010C 2D coordinate vision system while measuring the wear profile of the cutting edge of the planer knife.

A simple OCMM was used for optical measurements of the wear profile of the cutting edges of planer knives. It was a Quick Image QI–A2010C optical coordinate measuring machine from Mitutoyo (Kawasaki, Japan). Its basic technical characteristics are given in Table 2. The manufacturer’s website [32,33] presents a detailed description of such measurement systems. It is a system that can be treated as an optical coordinate measuring machine for 2D measurements or a coordinate vision system [34]. The QIPAK software (license number: 000131601) of this machine made it possible to register the wear profile of the tested blade in a 2D coordinate system. The resolution of the stage displacement in the X and Y directions was 0.1 µm. The measurement range in the X direction was 0–200 mm and in the Y direction 0–100 mm. Figure 7 shows the computer screen of this system during preparation for measuring the wear of the edge of the planer knife. However, Figure 8 shows a view of the measuring stage of the measuring system Quick Image QI–A2010C while examining the wear of the cutting edge of the planer knife.

Each file obtained from the optical measurements of the edge wear profile for planer knives numbered NN101 to NN106 contained approximately 4500 values recorded with a sampling step of 0.016 mm. The measurement time of one profile was approximately 2 min. However, for planer knives with numbers NN107 to NN112, 10,200 values were recorded for each knife with the same sampling step. The measurement time did not exceed 4 min. The measurement results were exported to a Microsoft Excel spreadsheet and processed using a program like the one used to process the results obtained during contact measurements. On this basis, as in the case of contact measurements, graphs of the wear of the cutting edges of the blades were prepared, and the values of the parameters *Mw*, *SVav*, and *Aw* characterizing the wear were determined.

## 3. Results and Discussion

**Figure 9:**
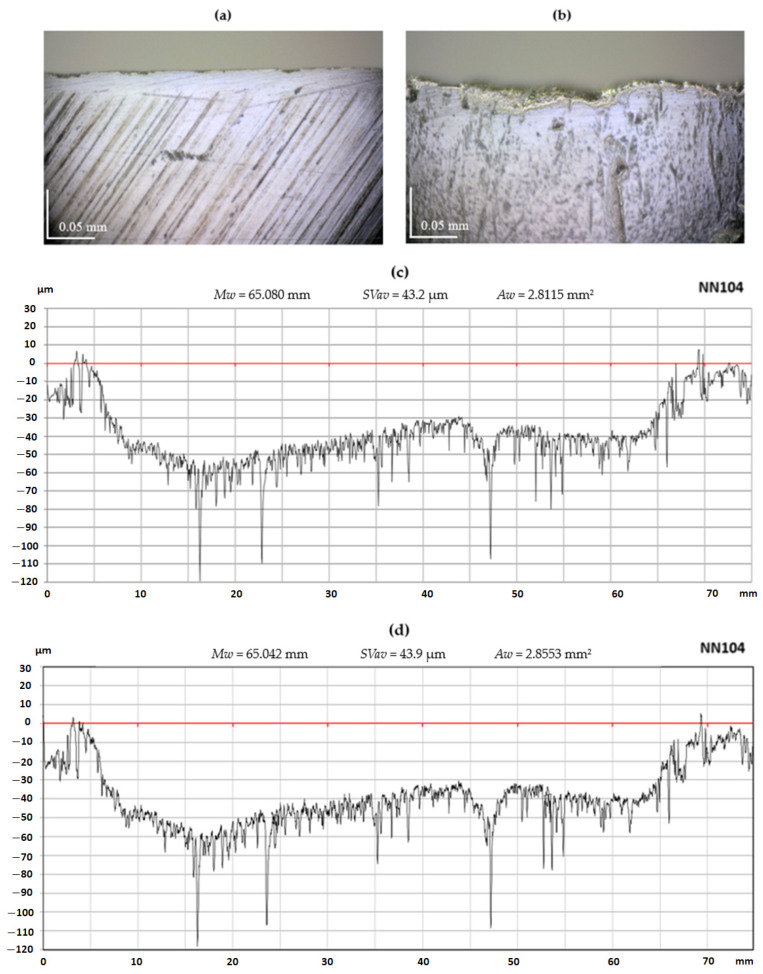
Photomicrographs and graphs depicting the test results of the NN104 planer knife: (**a**) a photo showing a view of the reference rake surface; (**b**) a photo showing the working rake surface; (**c**) a graph of the knife edge profile obtained from contact profilometry measurements, including wear parameters; and (**d**) a knife edge profile plot derived from OCMM measurement, including wear parameters.

**Figure 10:**
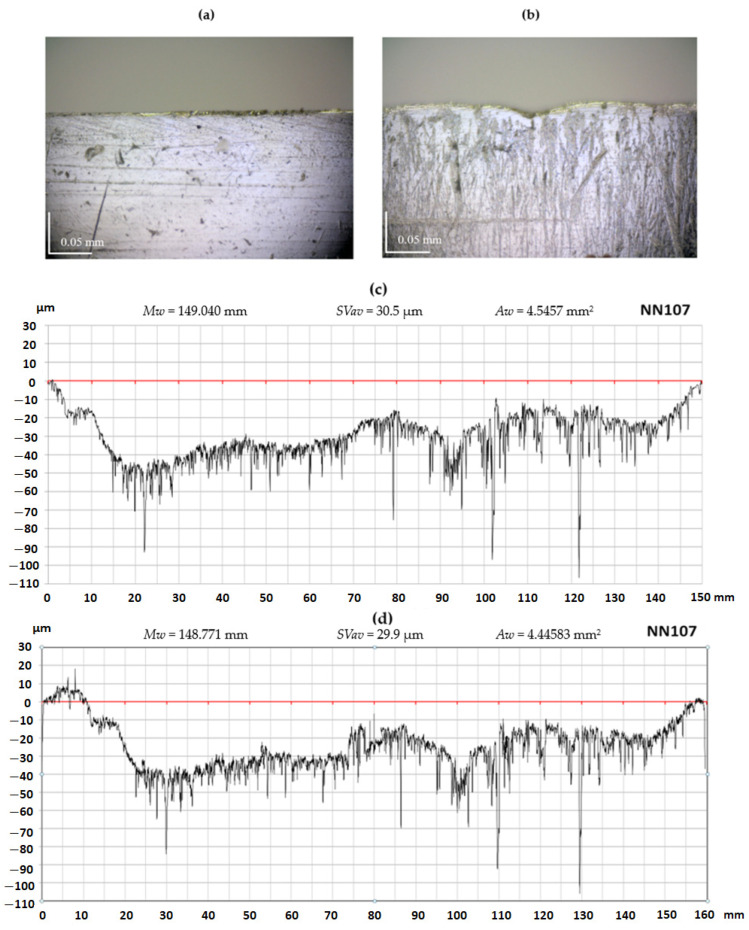
Photomicrographs and graphs depicting the test results of the NN107 planer knife: (**a**) a photo showing a view of the reference rake surface; (**b**) a photo showing the working rake surface; (**c**) a graph of the knife edge profile obtained from contact profilometry measurements, including wear parameters; and (**d**) a knife edge profile plot derived from OCMM measurement, including wear parameters.

Selected results of the measurement of the wear of planer knife blades obtained while experimental tests were carried out using a contact profilometer and a vision system are presented below. Figure 9c,d as well as Figure 10c,d include sample charts illustrating the wear of the cutting edges of the blades of the NN104 and NN107 planer knives obtained using the contact and vision methods, together with parameters characterizing the knife edges’ wear. A visual inspection of these graphs shows their high similarity, which means that the sensitivity and resolution of the vision system in the OCMM are adequate to determine the wear contour of the knife edge. To illustrate the process of the wear of the edges and rake surfaces of the blades, Figure 9a,b as well as Figure 9a,b show microscopic images of the rake surfaces of the blades of the NN104 and NN107 knives in the wear section and outside this zone. The rake surfaces of planer knives located outside the wear zone were treated as reference surfaces.

By analysing the outlines of the planer knife blade edge, shown in Figure 9c,d and Figure 10c,d, it is easy to see that the nominally straight edge of the blade suffered significant wear because of the operation. Locally, worn edge displacement reaches values of several dozen d. A comparison of the shape of the edge outlines of the planer knife blade visible in Figure 9 and Figure 10 allows us to conclude that the general form of the blade edge wear obtained from visual and contact measurements is similar. The values of the blade edge wear parameters are also very similar.

Upon careful comparison, it was observed that the graphs obtained using the optical method contained more details of the knife edge outline than the plots obtained using the contact method. This is because the optical method provided almost three times more signal samples to determine the edge outline, leading to a horizontal resolution of around three times greater than the contact method.

Based on the measurements carried out using the contact method and a coordinate vision system, the average depth of blade edge wear and the blade edge wear area were determined for all tested planer knives. The results of these measurements are summarized in Table 3 and Table 4, respectively.

Statistical tests were conducted to compare the edge wear measurements of the tested knives using both the contact and optical methods. This involved analysing wear profile histograms and assessing the measurement uncertainty of the average worn edge displacement *SVav*.

**Figure 11:**
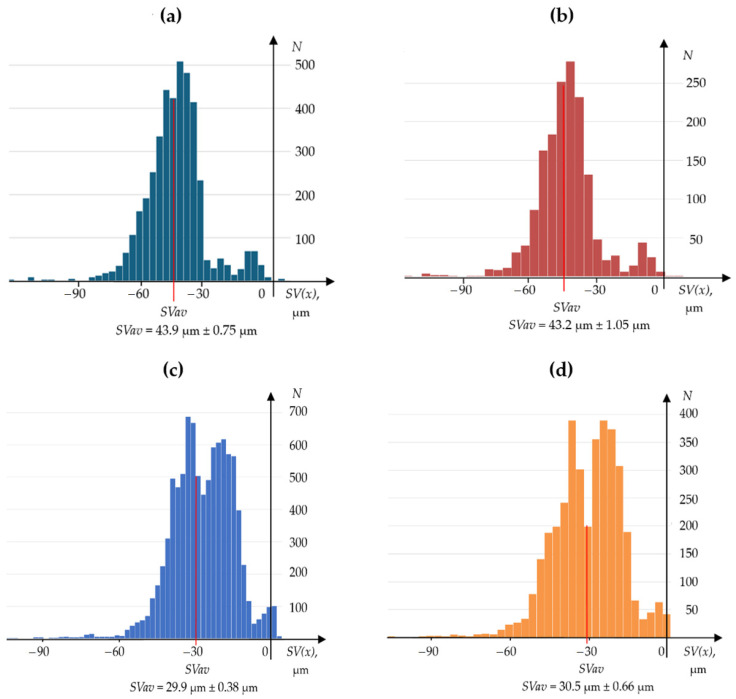
Histograms of the ordinate profiles of the worn cutting edges of the NN104 and NN107 planer knives, which were measured by the contact and optical methods (for profiles, see Figure 9 and Figure 10): a (**a**) histogram of the ordinates of the profile measured by the optical method for the NN 104 planer knife; (**b**) histogram of the ordinates of the profile measured by the contact method for the NN 104 planer knife; (**c**) histogram of the ordinates of the profile measured by the optical method for the NN 107 planer knife; and a (**d**) histogram of the ordinates of the profile measured by the contact method for the NN 107 planer knife.

Figure 11 shows histograms of the ordinates of the wear profiles of the NN101 and NN104 planer knives measured with the OCMM and the contact profilometer. Great similarity is visible between the histograms obtained for a given planer knife using both methods. The slight differences in the histograms are primarily due to the different number of *SV*(*x*) values obtained during the contact and optical measurements. The wear profiles measured with the OCMM contained more than twice as many data as the profiles measured with the contact profilometer. The reason for this was the difference in the sampling step of the measurement signal. The sampling step during the optical measurements was 0.016 mm, and during the contact measurements, it was 0.040 mm.

The number of data n of the wear profile affects the uncertainty of the measurements of the average value of worn edge displacement *SVav*. This results from relations (4) and (5) shown below:

(4) $$\sigma_{SVav}=\frac{\sigma_{SV}}{\sqrt{n}}$$

(5) $$U\left(SVav\right)=k{\cdot \sigma }_{SVav},$$

where *σ_SVav_*—standard deviation of the average value of ordinates *SV*(*x*), μm; *σ_SV_*—standard deviation of the average value of ordinates *SV*(*x*), μm; *n*—number of samples in the wear zone; *U*(*SVav*)—expanded uncertainty of the average value of ordinates *SV*(*x*), μm; *k*—coverage factor.

In addition to the histograms, Figure 11 presents the values of the *SVav* parameter and the values of the measurement uncertainty of this parameter. The expanded uncertainty values were determined using Equations (4) and (5), using the coverage factor *k* = 3. In the histogram graphs shown in Figure 11, the values of the *SVav* parameter are marked with a red line.

If there are significant differences between the measured wear of planer knives and their actual wear, this may lead to the rejection of the optical measurement method. However, the consistent and repeatable measurement results support the practical use of this method, for instance, in monitoring the quality of planer knives and their regeneration processes.

## 4. Conclusions

The wear of planer knives’ edges is typically examined and assessed using a specialized contact profilometer in laboratory conditions. However, this method is challenging to use in industrial settings. It requires specific conditions, such as low vibration levels, dust-free air, and stable temperature, and is difficult to automate. Additionally, it necessitates highly qualified personnel and is relatively time-consuming. After conducting this research, it is concluded that the optical coordinate measuring machine (OCMM) with a vision system can be useful to assess the wear of planer knife edges when the wear measures several micrometres or more. The measurements of edge wear parameters using the OCMM have high resolution and accuracy. Conducting wear measurements of planer knives using the OCMM is much simpler than similar tests using contact profilers.

A comparative analysis of the measurement results of the average depth of wear of the knives’ planer edges using both methods indicates that, in general, the differences in measurement results do not exceed a few percent. A similar conclusion can be drawn from the comparison of the measurement results of the wear surface area of knife blade edges. The reasons for the discrepancies in the measurement results of parameters characterizing blade wear, determined with a contact profilometer and the OCMM with the vision system, include, above all, a different method of homing the knife concerning the measurement system in the profilometer and the OCMM. Other reasons for the discrepancies include differences in the number of samples used for calculations and, in the case of profilometry measurements of blades whose edge length exceeds 100 mm, the need to perform several measurements and combine the obtained results into one outline of the knife edge. When using a contact profilometer, the setup of the measuring element takes time and requires accurate positioning for precise measurements. This is because the instrument has a limited vertical measuring range, and the chisel-shaped tip of the profilometer stylus is narrow.

When measuring blade edge wear with a contact profilometer, the measurement time is approximately 10 min. If the length of the wear zone of the tested knife exceeds the travel range of the profilometer stylus, the measurements must be performed several times. Then, the measurement time increases accordingly. Meanwhile, the time for measuring the edge wear of a single planer knife using the OCMM Quick Image QI-A2010C does not exceed 4 min. The maximum length of a planer knife that can be tested with this system is 200 mm.

We are starting to conduct future research to improve the automation of planer knife wear measurements using OCMMs. This will involve introducing measuring systems with CNC (computer numeric control) and updating software. The goal is to implement this method in industrial practice in an offline version. Additionally, we are exploring the possibility of using the existing system to monitor tool wear in other processes. Furthermore, we plan to develop a similar vision system for the real-time monitoring of planer knife wear.

The application of similar OCMMs and vision systems, especially those with automated controlled table CNC, will likely increase the automation of the process of assessing planer knife edge wear and shorten the assessment time. Moreover, limiting the operator’s influence on the measurement process will reduce measurement uncertainties. CNC-controlled OCMMs with vision systems can be very beneficial in assessing the quality and wear of planer knives in industrial applications.

## Figures and Tables

**Table 1:** Nominal chemical composition of steel HS6-5-2 (in wt.%).

C	Si	Mn	P	S	Cr	Mo	W	V
0.8–0.88	max 0.45	max 0.4	max 0.03	max 0.03	3.8–4.5	4.7–4.2	5.9–6.7	1.7–2.1

**Table 2:** Main parameters of OCMM Quick Image QI–A2010C.

Model		QI-A2010C
Measurement mode		High-resolution mode/Normal mode
View field		32 × 24 mm
Measuring range (X, Y axis)		200 × 100 mm
Travel range (Z axis)		100 mm
Accuracy	Measurement accuracy within the screen	High-resolution mode: ±5 µm/Normal mode: ±8 µm
	Repeatability within the screen (±2 σ)	High-resolution mode: ±1 µm/Normal mode: ±2 µm
	Measurement accuracy	±(5 + 0.08 *L*) µm *L*: measuring length, mm
Imaging device		3M pixel, Colour camera
Optical system	Magnific. (Telecentric Optical System)	0.2×
	Working distance	90 mm
	Depth of focus	High-resolution mode: ±0.6 mm/Normal mode: ±11 mm
Illumination		Transmitted light: Green LED telecentric illumination; Coaxial Light: White LED; Ring light: quadrant white LED

**Table 3:** A comparative summary of the results was obtained from the profilometer and OCMM when measuring the average value of the worn edge displacement of the planer knife.

	Average Values of Worn Edge Displacement *SVav*, μm											
Knife No.	NN101	NN102	NN103	NN104	NN105	NN106	NN107	NN108	NN109	NN110	NN111	NN112
Results from contact profilometer	21.2	29.8	29.7	43.2	18.2	19.5	30.5	13.5	30.7	32.8	32.7	19.3
Results from OCMM	20.9	25.7	28.5	43.9	16.3	18.4	29.9	14.5	32.1	30.8	30.8	20.2
Result difference (absolute value)	0.3	4.1	1.2	0.7	1.9	1.1	0.6	1.0	1.4	2.0	1.9	0.9

**Table 4:** A comparative summary of the results was obtained from the profilometer and OCMM when measuring the wear surface area of the planer knife edges.

	Wear Area of Cutting Edge Planer Knives *Aw*, mm^2^											
Knife No.	NN101	NN102	NN103	NN104	NN105	NN106	NN107	NN108	NN109	NN110	NN111	NN112
Results from contact profilometer	1.317	1.967	2.049	2.811	1.189	1.277	4.546	1.986	4.483	4.875	4.823	2.880
Results from OCMM	1.328	1.661	1.845	2.856	1.100	1.236	4.448	2.198	4.823	4.688	4.695	3.054
Result difference (absolute value)	0.011	0.306	0.204	0.045	0.089	0.041	0.098	0.212	0.340	0.187	0.128	0.174

## Data Availability

The raw data supporting the conclusions of this article will be made available by the authors on request.

## Nomenclature

ARM	automatic magnification reading
CMOS	complementary metal–oxide–semiconductor
CNC	computer numeric control
FLC	flexible LED control
HSS	high-speed steel
LED	light-emitting diode
OCMM	optical coordinate measuring machine
*b*	thickness of the planer knife, mm
*k*	coverage factor
*n*	number of samples in the wear section
*x* *min*	abscissa of the beginning of the wear section, mm
*x* *max*	abscissa of the end of the wear section, mm
*B*	length of the planer knife, mm
*Aw*	wear area of the cutting edge, mm^2^
*H*	height of the planer knife, mm
*L*	measuring length, mm
*Mw*	machining width (width of the wear zone of the cutting edge), mm
*N*	count of ordinates *SV*(*x*) in each histogram bin
*SVav*	average value of worn edge displacement, μm
*SV*(*x*)	worn edge displacement at *x* point, μm
*U*(*_SVav_*_)_	expanded uncertainty of the average value of ordinates *SV*(*x*), μm
*σ_SV_*	standard deviation of ordinates *SV*(*x*), μm
*σ_SVav_*	standard deviation of the average value of ordinates *SV*(*x*), μm
*Δx*	sampling step, mm
